# Surveillance as Determinant of Long-Term Survival in Non-Transplanted Hepatocellular Carcinoma Patients

**DOI:** 10.3390/cancers13040897

**Published:** 2021-02-20

**Authors:** Filippo Pelizzaro, Alessandro Vitale, Anna Sartori, Andrea Vieno, Barbara Penzo, Francesco Paolo Russo, Anna Chiara Frigo, Edoardo G Giannini, Manuela Piccinnu, Gian Ludovico Rapaccini, Maria Di Marco, Eugenio Caturelli, Marco Zoli, Rodolfo Sacco, Ciro Celsa, Fabio Marra, Andrea Mega, Maria Guarino, Antonio Gasbarrini, Gianluca Svegliati-Baroni, Francesco Giuseppe Foschi, Andrea Olivani, Alberto Masotto, Pietro Coccoli, Giovanni Raimondo, Francesco Azzaroli, Gianpaolo Vidili, Maurizia Rossana Brunetto, Franco Trevisani, Fabio Farinati, on behalf of ITA.LI.CA Study Group

**Affiliations:** 1 Gastroenterology Unit, Department of Surgery, Oncology and Gastroenterology, University of Padova, 35128 Padova, Italy; filippo.pelizzaro@unipd.it (F.P.); anna.sartori@aulss2.veneto.it (A.S.); andrea.vieno@studenti.univr.it (A.V.); barbara.penzo@aopd.veneto.it (B.P.); francescopaolo.russo@unipd.it (F.P.R.); 2 Hepatobiliary Surgery and Liver Transplantation Unit, Department of Surgery, Oncology and Gastroenterology, University of Padova, 35128 Padova, Italy; alessandro.vitale@unipd.it; 3 Biostatistics Unit, Department of Cardiac, Thoracic, Vascular Sciences and Public Health, University of Padova, 35128 Padova, Italy; annachiara.frigo@unipd.it; 4 Gastroenterology Unit, Department of Internal Medicine, University of Genova, IRCCS Policlinico San Martino, 16132 Genova, Italy; egiannini@unige.it; 5 Internal Medicine–Piscaglia Unit, Department of Medical and Surgical Sciences, IRCCS Azienda Ospedaliero-Universitaria di Bologna, 40126 Bologna, Italy; manuela.piccinnu@uniroma1.it; 6 Gastroenterology Unit, Fondazione Policlinico Universitario A. Gemelli, IRCCS, 00168 Roma, Italy; gianludovico.rapaccini@unicatt.it; 7 Medicine Unit, Bolognini Hospital, 24068 Seriate, Italy; maria.dimarco@bolognini.bg.it; 8 Gastroenterology Unit, Belcolle Hospital, 01100 Viterbo, Italy; e.caturelli@tiscalinet.it; 9 Internal Medicine–Zoli Unit, Department of Medical and Surgical Sciences, IRCCS Azienda Ospedaliero-Universitaria di Bologna, 40126 Bologna, Italy; marco.zoli@unibo.it; 10 Gastroenterology and Digestive Endoscopy Unit, Foggia University Hospital, 71122 Foggia, Italy; r.sacco@ao-pisa.toscana.it; 11 Gastroenterology & Hepatology Unit, Department of Health Promotion, Mother & Child Care, Internal Medicine & Medical Specialties, PROMISE, University of Palermo, 90133 Palermo, Italy; ciro.celsa@unipa.it; 12 Internal Medicine and Hepatology Unit, Department of Experimental and Clinical Medicine, University of Firenze, 50121 Firenze, Italy; fabio.marra@unifi.it; 13 Gastroenterology Unit, Bolzano Regional Hospital, 39100 Bolzano, Italy; andrea.mega@sabes.it; 14 Gastroenterology Unit, Department of Clinical Medicine and Surgery, University of Napoli “Federico II”, 80138 Napoli, Italy; maria.guarino@unina.it; 15 Internal Medicine and Gastroenterology Unit, Policlinico Gemelli, Università Cattolica del Sacro Cuore, 00168 Roma, Italy; antonio.gasbarrini@unicatt.it; 16 Gastroenterology Unit, Polytechnic University of Marche, 60121 Ancona, Italy; g.svegliati@univpm.it; 17 Department of Internal Medicine, Ospedale per gli Infermi di Faenza, 48018 Faenza, Italy; francesco.foschi@auslromagna.it; 18 Infectious Diseases and Hepatology Unit, Azienda Ospedaliero-Universitaria di Parma, 43126 Parma, Italy; aolivani@ao.pr.it; 19 Gastroenterology Unit, Ospedale Sacro Cuore Don Calabria, 37024 Negrar, Italy; alberto.masotto@sacrocuore.it; 20 Hepato-Gastroenterology Unit, Department of Clinical Medicine and Surgery, University of Napoli “Federico II”, 80138 Napoli, Italy; pietro.coccoli@unina.it; 21 Clinical and Molecular Hepatology Unit, Department of Clinical and Experimental Medicine, University of Messina, 98122 Messina, Italy; raimondo@unime.it; 22 Gastroenterology Unit, Department of Surgical and Medical Sciences, IRCCS Azienda Ospedaliero-Universitaria di Bologna, 40126 Bologna, Italy; francesco.azzaroli@aosp.bo.it; 23 Clinica Medica Unit, Department of Medical, Surgical and Experimental Sciences, University of Sassari, Azienda Ospedaliero-Universitaria di Sassari, 07100 Sassari, Italy; gianpaolovidili@uniss.it; 24 Department of Clinical and Experimental Medicine, Hepatology and Liver Physiopathology Laboratory and Internal Medicine, University of Pisa, 56126 Pisa, Italy; brunetto@med-club.com; 25 Medical Semeiotics Unit, Department of Medical and Surgical Sciences, IRCCS Azienda Ospedaliero-Universitaria di Bologna, 40138 Bologna, Italy; franco.trevisani@unibo.it

**Keywords:** hepatocellular carcinoma, long-term survival, surveillance, cancer stage, treatment

## Abstract

**Simple Summary:**

Some patients with hepatocellular carcinoma (HCC) obtain a very long survival, irrespective of any prediction. In this study, we looked for the impact of surveillance in long-term survival of HCC patients. After adjustment for confounders in multivariable logistic regression analysis, diagnosis under surveillance remained an independent predictor of long-term survival. In the surveillance group, observed and lead-time corrected survivals were significantly longer than in patients with casual/symptomatic diagnosis. However, when adjusted for baseline characteristics with inverse probability weights, surveillance and no surveillance groups demonstrated a similar survival, suggesting that the beneficial effect of surveillance is mediated by early stage diagnosis, which allows higher applicability of curative treatments. Surveillance is a major determinant of long-term survival and a wide implementation of surveillance programs should be pursued in order to improve the still poor prognosis of HCC patients.

**Abstract:**

Purpose: We aimed at assessing the impact of surveillance on long-term survival in HCC patients. Methods: From the ITA.LI.CA database, we selected 1028 cases with long (≥5 years, LS group) and 2721 controls with short-term survival (<5 years, SS group). The association between surveillance and LS was adjusted for confounders by multivariable logistic regression analysis. Survival of surveilled patients was presented both as observed and corrected for the lead-time bias, and the comparison of survival between surveillance and no surveillance groups was also performed after balancing the baseline characteristics with inverse probability weights (IPW). Results: LS patients were more frequently diagnosed under surveillance (*p* < 0.0001), and had more favorable baseline characteristics. Surveillance was an independent predictor of LS (OR = 1.413, 95% CI 1.195–1.671; *p* < 0.0001). The observed and the lead-time corrected survival of surveilled patients were significantly longer compared to the survival of not surveilled patients (*p* < 0.0001 and *p* = 0.0008, respectively). In IPW adjusted populations, no survival differences were demonstrated between the two groups (*p* = 0.30). Conclusions: Surveillance, increasing early-stage diagnosis and applicability of curative treatments, is a fundamental determinant of long-term survival in HCC patients. A wide implementation of surveillance programs should be pursued in order to improve HCC patients’ prognosis.

## 1. Introduction

Hepatocellular carcinoma (HCC) is a leading cause of cancer-related death worldwide [1]. According to the International Agency for Research on Cancer, incidence and mortality in 2018 involved 841,080 and 781,631 patients, respectively, with an age-standardized incidence rate of 9.3/100,000 and an age-standardized mortality of 8.5/100,000 [1]. This small difference could be explained by the low five-year survival rate of HCC patients (currently 12–14% in the United States) [2]. In Italy, despite the improvement of prognosis recently observed [3], the long-term survival rate remains around 20% [4].

The individual prognosis of HCC patients is however highly unpredictable and not always dismal. The great variability in survival is justified by the peculiar features of these patients in whom prognosis depends on several parameters, not only including tumor burden, liver functional reserve, and general conditions (characteristics incorporated in the most commonly used staging approach, the Barcelona Clinic Liver Cancer [BCLC] system [5]), but also tumor biology [6,7,8,9], gender [10], immunological response of the host [11], and therapeutic choices [12]. As a result, HCC patients may survive from a few months to many years. Studies looking for the predictors of long-term survival showed that early stage at diagnosis, preserved liver function and type of treatment performed are pivotal parameters in predicting a good prognosis [13,14,15]. With the aim of improving patients’ prognosis, by increasing early diagnosis and applicability of curative treatments, international guidelines recommend periodic surveillance in patients at risk of developing HCC [5,16]. These indications are supported by data deriving from two Chinese randomized controlled trials conducted in HBV-infected patients [17,18], several cohort studies [19,20,21,22,23,24], and meta-analysis [25,26]. Although a previous report indicate that the benefit of surveillance over no surveillance strategies is evident from the third year of follow-up [27], only limited data are currently available about the role of periodic screening in achieving a long-term survival. In this study, we aimed at evaluating the impact of surveillance on long-term survival in non-transplanted HCC patients.

## 2. Materials and Methods

In the Italian Liver Cancer (ITA.LI.CA) database, including 7816 HCC patients consecutively evaluated and managed from January 1987 to December 2018 in 24 participating Institutions, data are prospectively collected, updated every 2 years, and periodically revised by the ITA.LI.CA coordinator center (Semeiotics Unit, Alma Mater Studiorum-Bologna University).

**Figure 1:**
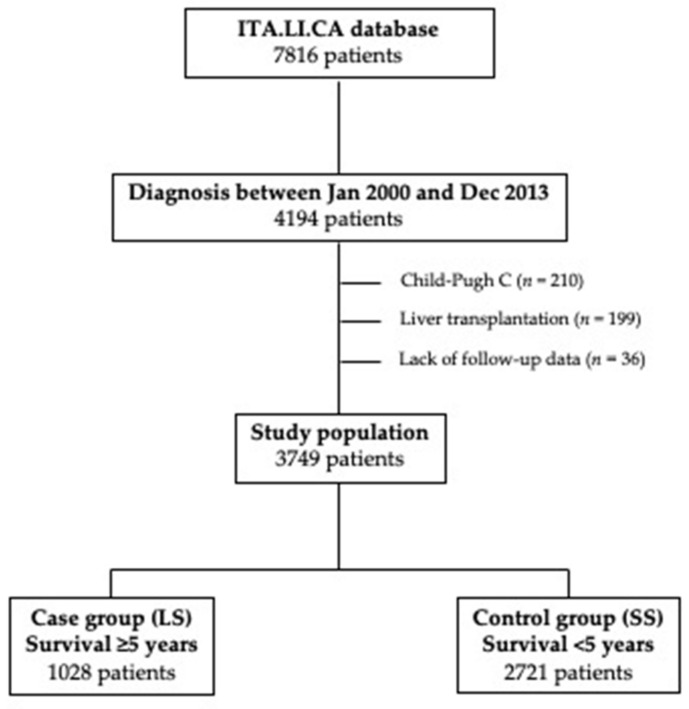
Study flow chart. Selection of patients finally included in the case (long-term survivors—LS) and control (short-term survivors—SS) groups.

From the ITA.LI.CA database, we selected the patients diagnosed with HCC from January 2000 to December 2013 (*n* = 4194). After the removal of 199 patients treated with liver transplantation (since transplant opens a peculiar scenario in terms of long-term survival), 210 Child-Pugh C patients (excluded from surveillance because advanced liver failure prevents effective HCC therapies), and 36 patients without survival data, in this study, 3749 patients were considered. Patients were divided in two groups according to their survival: 1028 patients (27.4%) showing a survival ≥5 years entered in the case group (long-term survivors, LS), while the remaining with a survival shorter than 5 years (*n* = 2721; 72.6%) were selected as controls (short-term survivors, SS) (Figure 1).

All patients included in this study fitted the criteria for entering in a surveillance program according to guidelines (cirrhotic patients in Child-Pugh classes A and B; non-cirrhotic HBV patients at intermediate or high risk of HCC; non-cirrhotic F3 patients perceived at high risk of tumor development) [5]. In the ITA.LI.CA database, the modality of HCC diagnosis (casual, achieved under surveillance, or as a consequence of the development of cancer-related symptoms) is recorded. In patients diagnosed under surveillance, data about the interval and the surveillance tests are also collected. Considering the nature of ITA.LI.CA database, surveillance protocols were not standardized across different Institutions. The interval of surveillance was established by the referring physician of each patient who was not necessarily one of the ITA.LI.CA clinicians, since a number of patients included in the database are referred to ITA.LI.CA Institutions after diagnosis for treatment purposes. Nevertheless, the six-months interval was the most frequently adopted among the patients included in the ITA.LI.CA database. As far as surveillance tests are considered, in all patients diagnosed under surveillance included in this study, the periodic repetition of liver ultrasonography was performed, with or without the adjunctive determination of alpha-fetoprotein (AFP) (left as a complementary choice of the clinician).

HCC diagnosis was histologically confirmed in 215 LS patients (20.9%) and in 468 SS patients (17.2%), whereas in the remaining cases, it was based on the typical features at imaging (i.e., at dynamic computed tomography or magnetic resonance), according to guidelines [5].

In the ITA.LI.CA database, the following standard demographic and clinical data are collected: Age, sex, comorbidities, body mass index (BMI), Eastern Cooperative Oncology Group performance status (ECOG-PS), general symptoms, modality of HCC diagnosis (unequivocal and radiological findings or biopsy/surgical specimens), etiology, serological parameters (albumin, bilirubin, INR, creatinine, sodium, platelet count, AFP), Child-Pugh score, Model for End-Stage Liver Disease (MELD) score, and clinically significant portal hypertension (CSPH). Tumor characteristics (location, size and number of nodules, macrovascular invasion [MVI], and extrahepatic spread [EHS]) and cause of death are also collected. CSPH diagnosis was based on unequivocal clinical signs (presence of esophageal varices, ascites, or splenomegaly and platelet count <100,000/mL), since hepatic venous gradients are not generally assessed [28].

Recently, the ITA.LI.CA staging system, externally validated [29,30], demonstrated the highest prognostic power compared to the other prognostic systems and was therefore considered in the present study.

Moreover, for the purpose of this paper, each ITA.LI.CA Institution was categorized, considering the volume of patients managed, in “low-” or “high-volume” centers, according to the average annual HCC case volume (below vs. above the median of the 24 centers, respectively).

From the therapeutic point of view, five groups were created: Liver resection (LR), ablation (ABL, including percutaneous ethanol injection, radiofrequency, and microwave ablation, either percutaneous or laparoscopic); intra-arterial therapies (IAT), systemic therapy with sorafenib (SOR), and “other” therapies (including best supportive care [BSC]). In patients managed with more than one treatment, only the more radical one (main treatment) was considered, according to the following hierarchy: LR, ABL, IAT, SOR, and OTHERS [12].

### Statistical Analysis

Categorical variables were expressed as absolute frequency and percentage, while continuous variables as medians and interquartile range (IQR). Quantitative data were compared with Student’s *t* test, while categorical variables with χ^2^ test and Fischer’s exact test, as appropriate.

A multiple logistic regression analysis was performed to identify independent predictors of LS, considering only the variables significantly or borderline (*p* ≤ 0.10) associated with survival in the univariate analysis. Since the aim of this study was to evaluate the impact of surveillance on long-term survival, multicollinearity analysis was performed. To exclude multicollinearity between surveillance and other variables, we analyzed tolerance (an indicator of how much collinearity that a regression analysis can tolerate) and variance inflation factor (an indicator of how much of the inflation of the standard error could be caused by collinearity) using a specific “collinearity diagnostics package” for STATA [31]. We also evaluated the calibration of the final model using the Calibration belt and test [32]. Finally, 1000 bootstrap replications of the final model were performed (reporting bootstrap standard errors and confidence intervals) to correct for optimism.

Survivals were expressed as median and 95% confidence interval (CI). Overall survival was calculated from HCC diagnosis to death, drop-out, or last follow-up visit, with data censored on 31 December 2018. The Kaplan–Meier method and the log-rank test were used to estimate and compare survival curves. Survival analyses were performed both before and after correction for the lead time bias in patients with HCC diagnosed under surveillance, as previously reported [27]. Moreover, in order to correct for all biases in the comparison between surveillance and no surveillance groups, propensity score values and inverse probability weights (IPW) were then calculated using generalized boosted models as described by McCaffrey et al. [33]. This is a machine learning technique using a flexible estimation method that can adjust for a large number of covariates. All potential confounders were included in boosted models: Sex, age, etiology, liver function, tumor related variables, radical treatment, and center volume. In order to reduce the type I error rate (because of the inflated sample size in the pseudo data), we used stabilized weights (SW) according the formula:

SW = *p*/PS

for the study group,

SW = (1 − *p*)/(1 − PS)

for the control group, where *p* is the probability of etiology without considering covariates and PS is the propensity score.

Finally, weighted survival curves were calculated using the Kaplan–Meier method and compared using the Log Rank test.

Missing data of study covariates always involved less than 10% of patients. Thus, they were estimated using the Maximum Likelihood Estimation method [34].

In all analyses, a two-tailed *p*-value < 0.05 was considered statistically significant. All analyses were performed in JMP^®^ 9.0.1 package (1989–2010 SAS Institute Inc., Cary, NC, USA), STATA13.0 (Copyright 1985–2013 StataCorp LP, College Station, TX, USA), and R. app 4.0.0 GUI 1.71 (S. Urbanek & H.-J. Bibiko, © R Foundation for Statistical Computing, 2016).

## 3. Results

### 3.1. Patients’ Characteristics

The median follow-up was 92.3 months (95% CI 89.2–94.0) in LS group and 19.0 months (18.0–20.0) in SS group. During the follow-up, 470 patients (45.7%) in LS group died, 166 (35.3%) from tumor progression, 73 (15.5%) from liver failure, 160 (34.1%) from other causes, and 71 (15.1%) from unspecified causes. All SS patients were dead at the end of the follow-up, with tumor progression being the most frequent cause (1118 patients, 41.1%), followed by liver failure (331, 12.1%), other causes (1006, 37.0%), and not reported causes (266, 9.8%).

The median overall survival (OS) was 120.0 months (95% CI 109.7–130.3) in LS patients and 19.0 months (95% CI 18.1–19.9) in SS patients (*p* < 0.0001).

Baseline characteristics of LS and SS patients are shown in Table 1. Cases and controls were comparable for gender, presence of type 2 diabetes mellitus and viral etiology. LS patients were slightly younger than SS patients (*p* = 0.04) and showed a significantly higher prevalence of overweight (35.7% vs. 27.3%), but the two groups were comparable in the prevalence of metabolic disfunction-associated fatty liver disease (MAFLD) (14.2% vs. 12.0%, respectively; *p* = 0.08). LS patients showed a higher prevalence of HCC developed on a non-cirrhotic liver (8.3% vs. 5.0%; *p* = 0.0002) and a lower prevalence of CSPH (72.0% vs. 83.2%, *p* < 0.0001). Liver function was better preserved in LS than in SS patients (Child-Pugh class A in 86.9% and 68.0%, and median MELD score of 9 [7,8,9,10] and 10 [8,9,10,11,12], respectively; *p* < 0.0001 in both cases).

LS and SS patients significantly differed in terms of diagnosis under surveillance (67.9% vs. 55.7%; *p* < 0.0001). The median duration of surveillance was 48.0 months (IQR, 16.0–120.0) in SS and 60.0 months (IQR, 24.0–120.0) in LS patients (*p* = 0.06). Of the patients included in this study, 1539 (69.5%) underwent semiannual surveillance (76.2% in LS and 65.7% in SS group), 266 (12.0%) annual surveillance (9.8% in LS and 12.9% in SS groups), and 330 (14.9%) were followed-up with a three-month schedule (12.0% in LS and 16.2% in SS group). Other surveillance intervals were less frequently adopted.

As far as oncological variables are concerned, LS patients showed better preserved clinical conditions (ECOG-PS 0 in 86.9% vs. 69.1%; *p* < 0.0001), lower number (*p* < 0.0001) and size (*p* < 0.0001) of nodules, lower prevalence of MVI (3.4% vs. 15.9%; *p* < 0.0001), EHS (0.8% vs. 4.7%; *p* < 0.0001), and AFP levels (≤200 ng/mL in 79.4% vs. 66.2%; *p* < 0.0001). Early-stage tumor, according to ITA.LI.CA classification, were more frequently diagnosed in LS patients (stages 0–A in 67.7% of LS and in 33.6% of SS patients).

Lastly, considering the main treatment, LS patients more frequently underwent LR (29.3% vs. 11.4%) and ABL (47.4% vs. 27.8%), and less frequently IAT (13.4% vs. 27.3%), SOR (1.1% vs. 6.1%) and BSC or other treatments (8.8% vs. 27.4%). Eight hundred and eighty-eight patients (23.7%) were managed in “low-volume” centers and 2861 patients (76.3%) in “high-volume” Institutions, with a significantly higher prevalence of LS than of SS patients managed in “low-volume” hospitals (32.9% vs. 20.2%; *p* < 0.0001).

### 3.2. Multivariable Logistic Regression Analysis

In addition to diagnosis under surveillance (odds ratio [OR] = 1.681, 95% CI 1.445–1.956; *p* < 0.0001), several other variables resulted associated (*p* ≤ 0.10) with the survival group at the univariate logistic regression analysis: Age, overweight, cirrhosis, presence of MAFLD, ECOG-PS, CSPH, MELD score, Child-Pugh class, multifocality, tumor size, MVI, EHS, AFP, ITA.LI.CA stage, “volume” of the ITA.LI.CA Institution, and main treatment. Considering that the aim of this study was to determine the impact of surveillance on long-term survival, we performed a multicollinearity analysis in order to exclude from the multivariable model variables collinear with surveillance. The final model obtained is described in Table 2. Diagnosis under surveillance remained independently associated with long-term survival (adjusted OR = 1.413, 95% CI 1.195–1.671; *p* < 0.0001). Other variables significantly associated with LS were lower age, presence of MAFLD, absence of CSPH, lower MELD score, and being managed in low-volume centers. As expected, the variable with the strongest independent impact on long-term survival was main treatment: Curative therapies (LR + ABL) were associated with an OR of long-term survival of 3.924 (95% CI 3.312–4.650; *p* < 0.0001).

The results of the calibration test (Figure S1; statistic = 0.09, *p* = 0.76) suggest that the hypothesis of good calibration of the final model is not rejected (at the classically adopted 0.05 level). Similar conclusions can be drawn from the interpretation of the produced plot (calibration belt), reported in the Figure S1. We note that both the 80% and 95% calibration belts encompass the bisector over the whole range of the predicted probabilities. This suggests that the predictions of the model do not significantly deviate from the observed rate in the training sample (which means that the model’s internal calibration is acceptable). Moreover, bootstrap standard errors and confidence intervals (Table S1) overlapped with that of the final model in Table 2, suggesting that the final model doesn’t suffer of optimistic bias.

### 3.3. Survival Analysis

**Figure 2:**
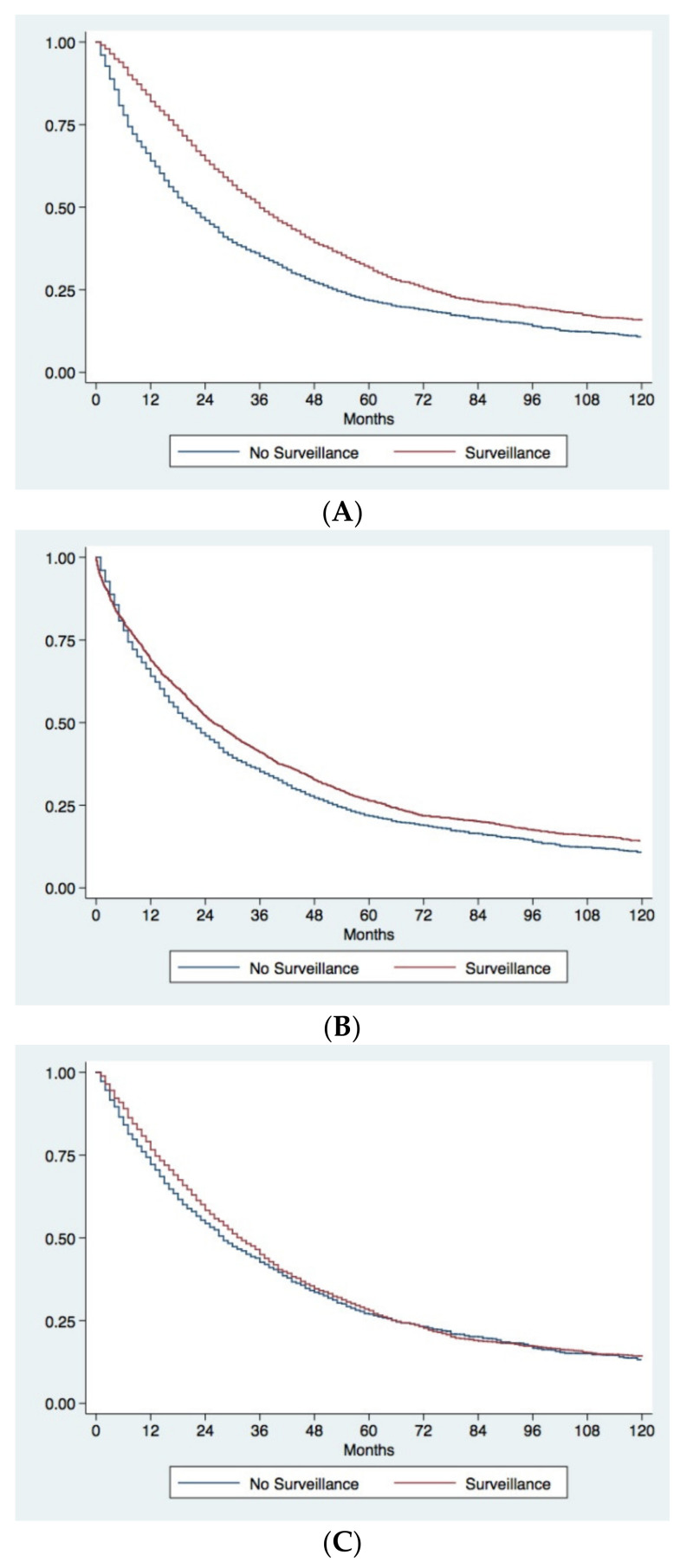
Kaplan–Meier survival curves comparing surveillance and no surveillance groups. (**A**) Observed survival of patients diagnosed under surveillance or with a casual/symptomatic diagnosis. Patients diagnosed under surveillance demonstrated a significantly longer survival (*p* < 0.0001). (**B**) Observed survival of patients with casual/symptomatic diagnosis compared to corrected survival in surveilled patients. Surveillance significantly improves prognosis of patients even after correction for the lead-time bias (*p* = 0.0008). (**C**) Comparison of survival between surveilled and not surveilled patients after adjustment for adjustment for confounders with IPW. The two groups of patients showed similar survival (*p* = 0.30).

The unadjusted Kaplan–Meier analysis demonstrated a considerable survival advantage in patients diagnosed under surveillance compared to patients diagnosed incidentally or because the development of symptoms. Surveilled patients had a median OS of 36.0 months (95% CI 33.9–38.1) compared to 20.0 months (95% CI 18.0–22.0) in not-surveilled patients, with five-year survival rates of 31.5% and 21.5%, respectively (*p* < 0.0001) (Figure 2A). Even after correction for lead-time bias, surveillance remained associated with a better prognosis. The median survival of surveilled patients corrected for the lead-time bias was 25.6 months (95% CI 23.6–27.5), with a five-year corrected survival rate of 26.3%. These figures were again significantly higher than those observed in not-surveilled patients (*p* = 0.0008) (Figure 2B).

In order to correct for all biases in the comparison between surveillance and no surveillance groups, an IPW analysis was performed. Baseline characteristics of surveillance and no surveillance groups before and after IPW are showed in Table 3. Before IPW, in the surveillance group, there was a significant lower percentage of males, of patients with BMI >25 kg/m^2^, with type 2 diabetes mellitus and MAFLD, and a significantly higher percentage of cirrhotics, with a virus-related liver disease and CSPH. Surveilled patients had a better preserved liver function (Child-Pugh A in 76.6% vs. 68.0%; *p* < 0.0001) and better clinical conditions (ECOG-PS 0 in 81.3% vs. 63.5%; *p* < 0.0001). As far as oncological variables were concerned, surveilled patients presented an overall lower tumor burden and significantly lower levels of AFP. Finally, a significant higher proportion of patients diagnosed during surveillance underwent to LR or ABL. After IPW, two populations absolutely comparable in all the baseline characteristics were obtained (Table 3). The survival analysis performed in the two IPW adjusted populations demonstrated no differences in prognosis between surveilled and not surveilled groups (median OS in surveilled group 31.0 months [95% CI 30.0–33.0] vs. 28.0 months [95% CI 26.0–30.0] in not surveilled patients; five-year survival rates 28.0% and 27.0% respectively; *p* = 0.30) (Figure 2C).

## 4. Discussion

Several attempts to establish the HCC prognosis, in both untreated and treated patients have been made so far, also with the aim of determining the actual survival benefit of each treatment in each cancer stage [7,10,12,29,35,36,37,38]. In untreated patients, for instance, median OS has been reported to range from 25–38 months in BCLC stages 0–A and to be of 6 months in BCLC D [10]. The amenability to the most effective treatment, defined on an individualized basis, is an additional relevant factor that increase the prognostic variability among patients [12]. In this respect, it is worth noting that LR achieves a net survival benefit over loco-regional treatments across different BCLC stages [39]. Nevertheless, the indicated treatment may be not always prescribed or available, even in wealthy countries [40]. Beyond that, the survival of HCC patients can be unexpectedly long, or short, irrespective of what can be foreseen considering baseline clinical characteristics and treatment received, since the biologic aggressiveness of the tumor and the immunologic defenses of the host play a crucial role in determining the treatment outcome [6,7,8,9,11].

Some studies tried to clarify the factors associated with long-term survival in different therapeutic settings. Following LR, tumor diameter, presence of single node, and absence of microvascular invasion [13,14], as well as absence of cirrhosis [15], independently predict a very long survival. Other studies focused on the prediction of the outcome after ABL [41,42], IAT [43,44], or systemic therapies [45,46]. However, for unselected HCC patients, models based on routinely available clinical characteristics capable to predict long-term survival without liver transplant are still lacking.

Beyond that, in the prognostic stratification of HCC patients, surveillance is an important parameter that has to be considered. Although only two randomized controlled trials have ever been conducted on this topic [17,18], several cohort studies [19,20,21,22,23,24] and meta-analyses [25,26] showed that surveillance is associated with a better prognosis. As a matter of fact, all the major international guidelines recommend surveillance in patients at risk of developing HCC, with the aim of maximizing survival probabilities, achieving an early diagnosis which allows the applicability of potentially curative treatments [5,16]. In the literature, some data demonstrate that surveillance strategies exert their benefit on survival depending on the length of follow-up. The survival benefit provided by surveillance over casual/symptomatic diagnosis become factual for long follow-up (i.e., after the third year), with the short-term survival advantage being largely attributable to lead-time bias [27]. However, the actual role of surveillance in achieving a long-term survival is still not defined.

Bearing this in mind, we aimed to evaluate the impact of surveillance on long-term survival comparing a group of non-transplanted HCC patients showing a survival ≥5 years with a group of contemporaneous patients with shorter survival. As expected, LS patients showed favorable baseline characteristics in terms of severity of liver disease (lower rates of CSPH, better Child-Pugh class and lower MELD score levels), clinical conditions (better ECOG-PS), and tumor burden (fewer and smaller nodules, less frequent MVI and EHS presence, lower levels of AFP). Overall, cancer stage at diagnosis was significantly earlier in LS patients and this, in addition to better preserved liver function and clinical conditions, allowed a higher applicability of curative treatments (LR and ABL). Concerning death causes, despite that a higher proportion of death for HCC progression could be expected in SS group, about 35% of LS patients eventually died from late tumor recurrence, without differences between cases and controls. Only less than half of LS patients (45.7%) were dead at the end of follow-up and this could have influenced this result. However, even patients with long survival after curative therapies persist at risk of recurrence and progression, with the five-year recurrence rates after LR being around 70% [5].

Although in both LS and SS groups a relatively high percentage of patients (more than 50%) was diagnosed under surveillance, in the former group, surveilled patients were significantly more represented (67.9% vs. 55.7%; *p* < 0.0001). Despite the fact that these figures substantially differ from other experiences published in the literature, which reported <20% of cirrhotics undergoing surveillance [47,48], the percentage of surveilled patients in this study is in line with previous works of the ITA.LI.CA group [3,49].

After correction for confounders, excluding from the multivariable model collinear variables, surveillance maintained an independent association with long-term survival. Other variables independently associated with long-term survival were younger age, absence of CSPH, and preserved liver function (lower MELD score). In addition, MAFLD, compared to other etiologies, proved to be associated with better prognosis. An intriguing result, that may seem counterintuitive, is the lower probability of long-term survival for patients managed in “high-volume” centers. We can speculate that this reflects the referral of patients more complex and “difficult to treat” to high-volume tertiary centers, as already demonstrated in other liver diseases, such as in primary sclerosing cholangitis [50].

Treatment emerged as a fundamental prognostic variable in the multivariable logistic regression analysis, with radical therapies (LR and ABL) being the strongest predictors of a better prognosis. Our data fuel the debated issue of HCC treatment. The BCLC system, endorsed by the European and American Guidelines [5,51], relies on a “stage hierarchy” philosophy, which recommends a specific treatment for each stage [52]. However, numerous studies report a poor adherence to its therapeutic indications [53,54,55], and several data show that curative therapies are superior to the standard of care in selected intermediate or advanced patients [52]. The so-called “therapeutic hierarchy” approach, which indicates a sequence of HCC treatments hierarchically organized according to their proven effectiveness (survival benefit), is now gaining ground as a strategy well in line with the evolving concept of “precision medicine”, i.e., a patient-tailored rather than a stage-dictated management [52].

In this study, as already demonstrated [19,20], diagnosis under surveillance proved to be associated with a better prognosis compared to casual/symptomatic diagnosis in the unadjusted survival analysis. Our study, as all cohort studies on surveillance performance, may suffer from length-time and lead-time biases [27,56]. Surveillance preferentially detects tumors with slow growth (length-time bias), and it may be possible that a higher percentage of aggressive HCC is present in SS group. However, in this study, the confounding effect of length-time bias was minimized by keeping in the surveillance group the patients in whom HCC diagnosis was anticipated (with respect to the scheduled surveillance test) due to the development of symptoms [56]. Although the lead-time bias loses most of its importance in long-surviving patients [27], we also accounted for its confounding effect in this study, correcting the survival of surveilled patients for the calculated lead-time. Surveillance maintained its prognostic benefit over casual/symptomatic diagnosis even after this correction. It can be speculated that patients who adhere to a regular surveillance schedule have also a higher compliance to the entire diagnostic and therapeutic process, thus improving their prognosis. Nevertheless, in order to account for all potential confounders, survival of surveillance and no surveillance groups were compared after adjustment for baseline characteristics with IPW. In these populations, the survival benefit of surveillance disappeared. This is reasonable because the benefit of surveillance relies not on an intrinsic property of the modality of diagnosis, but derives from the ability of periodic screening to detect HCC at an early stage and, in turn, increase the proportion of patients amenable to effective treatments. Therefore, in groups adjusted for baseline oncologic and therapeutic variables, surveillance lost its association with better prognosis. In any case, our findings support once more the recommendation of a widespread use of surveillance in all patients at risk for HCC, despite the lack of randomized controlled trials in cirrhotics and HCV patients with advanced fibrosis [5,57].

Despite the attempt made to minimize all confounding factors, the retrospective nature of our study makes it vulnerable to several unintended biases. However, we feel that the limitations of this study are overweighted by its strengths, among which the adjustment only for factors not collinear with surveillance in the multivariable logistic regression model to evaluate its independent prognostic role. Moreover, the survival benefit of surveillance was firstly adjusted for the lead-time bias and subsequently tested in populations balanced with IPW. We believe that our results strengthen the pivotal role of surveillance as prognostic predictor and further underlines the need to develop extensive screening programs and to foster a high adherence, in order to improve HCC patients’ prognosis through early diagnosis and delivery of curative treatments.

## 5. Conclusions

In addition to well-known predictors of survival, regular surveillance of patients at risk is a fundamental parameter that must be considered in the aim of achieving a long-term survival. Surveillance benefit are driven by an increase in early stage tumor detection and amenability to potentially curative treatments. Our results further and strongly underline the importance of implementing surveillance programs in all patients at risk of developing HCC.

## Figures and Tables

**Table 1:** Baseline characteristics of cases (long-term survivors—LS) and controls (short-term survivors—SS).

Variable		Cases—LS *n* = 1028	Controls—SS *n* = 2721	*p* ^†^
Gender - males		791 (76.9)	2067 (76.0)	0.55
Age (years)		69 (62–74)	69 (62–75)	0.04
BMI (kg/m^2^)				
	≤25	661 (64.3)	1977 (72.7)	<0.0001
	25–30	264 (25.7)	532 (19.5)	
	>30	103 (10.0)	212 (7.8)	
T2DM		339 (33.0)	891 (32.7)	0.91
Cirrhosis		943 (91.7)	2586 (95.0)	0.0002
Viral etiology		712 (69.3)	1899 (69.8)	0.75
MAFLD		146 (14.2)	327 (12.0)	0.08
CSPH		740 (72.0)	2263 (83.2)	<0.0001
Child-Pugh class				
	A	893 (86.9)	1850 (68.0)	<0.0001
	B	135 (13.1)	871 (32.0)	
MELD		9 (7–10)	10 (8–12)	<0.0001
Surveillance		698 (67.9)	1516 (55.7)	<0.0001
ECOG-PS 0		893 (86.9)	1880 (69.1)	<0.0001
Multifocality		257 (25.0)	1476 (54.2)	<0.0001
Number of nodules		1 (1–2)	2 (1–4)	<0.0001
Diameter (cm)		2.7 (2.0–3.7)	3.5 (2.3–5.3)	<0.0001
MVI		35 (3.4)	432 (15.9)	<0.0001
EHS		8 (0.8)	128 (4.7)	<0.0001
AFP ≤ 200 ng/mL		816 (79.4)	1802 (66.2)	<0.0001
ITA.LI.CA staging system				
	A	431 (42.0)	582 (21.4)	<0.0001
	B1	198 (19.3)	701 (25.8)	
	B2	59 (5.7)	290 (10.6)	
	B3	29 (2.8)	233 (8.6)	
	C	27 (2.6)	310 (11.4)	
	D	19 (1.8)	273 (10.0)	
Main treatment				
	LR	301 (29.3)	310 (11.4)	<0.0001
	ABL	487 (47.4)	757 (27.8)	
	IAT	138 (13.4)	743 (27.3)	
	SOR	11 (1.1)	166 (6.1)	
	Other	91 (8.8)	745 (27.4)	
Management in “Low-volume” Institutions		338 (32.9)	550 (20.2)	<0.0001

Continuous data are presented as median and interquartile range, while categorical variables are expressed as absolute frequency and percentage. † Student’s *t* test, χ^2^ test or Fischer’s exact test, as appropriate. Abbreviations: LS, long-term survivors; SS, short-term survivors; BMI, body mass index; T2DM, type 2 diabetes mellitus; MAFLD, metabolic defunction associated fatty liver disease; CSPH, clinically significant portal hypertension; MELD, Model for End Stage Liver Disease; ECOG-PS, Eastern Cooperative Oncology Group performance status; MVI, macrovascular invasion; EHS, extra-hepatic spread; AFP, alpha-fetoprotein; ITA.LI.CA, Italian Liver Cancer; LR, liver resection; ABL, ablation; IAT, intra-arterial therapy; SOR, sorafenib.

**Table 2:** Univariate and multivariate logistic regression analysis for independent predictors of long-term survivors (LS) group membership.

Variable		Univariable Analysis		Multivariable Analysis	
		OR (95% CI)	*p*	aOR (95% CI)	*p*
Surveillance	No	Ref	-	Ref	-
	Yes	1.681 (1.445–1.956)	<0.0001	1.413 (1.195–1.671)	<0.0001
Gender	Female	Ref	-		
	Male	0.947 (0.799–1.122)	0.53		
Age ^†^		0.993 (0.986–0.999)	0.04	0.989 (0.982–0.997)	0.008
BMI (kg/m^2^)	≤25	Ref	-		
	>25	1.475 (1.266-.719)	<0.0001		
T2DM	No	Ref	-		
	Yes	1.011 (0.867–1.177)	0.89		
Cirrhosis	No	Ref	-		
	Yes	0.579 (0.437–0.767)	<0.0001		
Viral Etiology	No	Ref	-		
	Yes	0.975 (0.835–1.140)	0.75		
MAFLD	No	Ref	-	Ref	-
	Yes	1.212 (0.983–1.495)	0.07	1.299 (1.032–1.636)	0.03
CSPH	No	Ref	-	Ref	-
	Yes	0.520 (0.439–0.616)	<0.0001	0.705 (0.582–0.854)	0.0003
Child-Pugh	A	Ref	-		
	B	0.321 (0.263–0.391)	<0.0001		
MELD ^†^		0.840 (0.816–0865)	<0.0001	0.877 (0.850–0.905)	<0.0001
ECOG-PS	0	Ref	-		
	≥1	0.338 (0.277–0.412)	<0.0001		
Multifocality	No	Ref	-		
	Yes	0.281 (0.240–0.330)	<0.0001		
Diameter (cm)	≤5	Ref	-		
	>5	0.325 (0.261–0.405)	<0.0001		
MVI	No	Ref	-		
	Yes	0.187 (0.131–0.266)	<0.0001		
EHS	No	Ref	-		
	Yes	0.159 (0.077–0.326)	<0.0001		
AFP (ng/mL)	≤200	Ref	-		
	>200	0.509 (0.429–0.604)	<0.0001		
ITA.LI.CA stage	0–A	Ref	-		
	B–D	0.241 (0.207–0.281)	<0.0001		
Treatment	Palliative	Ref	-	Ref	-
	Curative	4.810 (4.083–5.667)	<0.0001	3.924 (3.312–4.650)	<0.0001
ITA.LI.CA Institution	HV	Ref	-	Ref	-
	LV	1.934 (1.647–2.270)	<0.0001	1.741 (1.463–2.070)	<0.0001

^†^ In univariate and multivariate analysis age and MELD were considered as continuous variables. Palliative treatment: IAT, SOR and other; curative treatments: LR and ABL. Abbreviations: OR, Odds Ratio; CI, confidence interval; aOR, adjusted Odds Ratio; Ref, reference group; BMI, body mass index; T2DM, type 2 diabetes mellitus; MAFLD, metabolic disfunction associated fatty liver disease; CSPH, clinically significant portal hypertension; MELD, Model for End-Stage Liver Disease; ECOG-PS, Eastern Cooperative Oncology Group performance status; MVI, macrovascular invasion; EHS, extrahepatic spread; AFP, alpha-fetoprotein; ITA.LI.CA, Italian Liver Cancer; HV, high-volume institutions; LV, low-volume institutions.

**Table 3:** Baseline characteristics of surveillance and no surveillance groups before and after inverse probability weights.

Variable		Before IPW			After IPW		
		Surveillance (*n* = 2214)	No Surveillance (*n* = 1535)	*p* ^†^	Surveillance (*n* = 2215)	No Surveillance (*n* = 1531)	*p* ^†^
Gender—males		1621 (73.2)	1237 (80.6)	<0.0001	1676 (75.7)	1158 (75.7)	0.97
Age—≤70 years		1250 (56.5)	859 (56.0)	0.76	1228 (55.5)	853 (55.7)	0.95
BMI >25 kg/m^2^		621 (28.0)	490 (31.9)	0.01	627 (28.3)	451 (29.4)	0.46
T2DM		681 (30.8)	549 (35.8)	0.002	708 (32.0)	505 (33.0)	0.52
Cirrhosis		2140 (96.7)	1389 (90.5)	<0.0001	2089 (94.3)	1443 (94.2)	0.94
Viral etiology		1723 (77.8)	888 (57.8)	<0.0001	1544 (69.7)	1062 (69.4)	0.83
MAFLD		199 (9.0)	274 (17.8)	<0.0001	260 (11.7)	194 (12.6)	0.39
CSPH		1833 (82.8)	1170 (76.2)	<0.0001	1792 (80.9)	1235 (80.7)	0.90
Child-Pugh A		1699 (76.7)	1044 (68.0)	<0.0001	1606 (72.5)	1106 (72.2)	0.82
MELD >10		845 (38.2)	591 (38.5)	0.84	848 (38.3)	584 (38.1)	0.97
ECOG-PS 0		1799 (81.3)	974 (63.5)	<0.0001	1641 (74.1)	1129 (73.8)	0.82
Multifocality		852 (38.5)	881 (57.4)	<0.0001	1025 (46.3)	708 (46.2)	1.00
Diameter >5 cm		207 (9.4)	597 (38.9)	<0.0001	475 (21.4)	331 (21.6)	0.90
MVI		155 (7.0)	312 (20.3)	<0.0001	290 (13.1)	197 (12.9)	0.88
EHS		35 (1.6)	101 (6.6)	<0.0001	86 (3.9)	56 (3.7)	0.93
AFP ≤ 200 ng/mL		1628 (73.5)	990 (64.5)	<0.0001	1559 (70.4)	1073 (70.1)	0.83
ITA.LI.CA stage							
	0–A	1184 (53.5)	426 (27.8)	<0.0001	944 (42.6)	652 (42.5)	0.95
	B–D	1030 (46.5)	1109 (72.2)		1271 (57.4)	880 (57.5)	
Treatment							
	LR + ABL	1299 (58.7)	602 (39.2)	<0.0001	1120 (50.6)	771 (50.4)	0.89
	IAT + SOR + Other	915 (41.3)	933 (60.8)		1094 (49.4)	760 (49.6)	
“Low-volume” Institutions		543 (24.5)	345 (22.5)	0.15	537 (24.2)	374 (24.4)	0.88

^†^ Student’s *t* test, χ^2^ test or Fischer’s exact test, as appropriate. Abbreviations: IPW, inverse probability weights; BMI, body mass index; T2DM, type 2 diabetes mellitus; MAFLD, metabolic associated fatty liver disease; CSPH, clinically significant portal hypertension; MELD, Model for End Stage Liver Disease; ECOG-PS, Eastern Cooperative Oncology Group performance status; MVI, macrovascular invasion; EHS, extra-hepatic spread; AFP, alpha-fetoprotein; ITA.LI.CA, Italian Liver Cancer; LR, liver resection; ABL, ablation; IAT, intra-arterial therapy; SOR, sorafenib.

## Data Availability

The authors confirm that the data supporting the findings of this study are available within the article and its Supplementary Materials.

## Supplementary Materials

The following are available online at https://www.mdpi.com/2072-6694/13/4/897/s1, Figure S1: Calibration belt and test of the final multivariable logistic regression model, Table S1: Bootstrap standard errors and confidence intervals of our multivariable logistic model.
