# Supplementary material for: Surveillance as Determinant of Long-Term Survival in Non-Transplanted Hepatocellular Carcinoma Patients
Source: Cancers (Basel). 2021 Feb 20;13(4):897. doi: 10.3390/cancers13040897 (PMC7924561; doi:10.3390/cancers13040897)
Supplement: Supplementary file 1 [file cancers-13-00897-s001.pdf]

cancers MDPI

Supplementary Material: Surveillance as Determinant of Long-Term Survival in Non-Transplanted Hepatocellular Carcinoma Patients

Filippo Pelizzaro, Alessandro Vitale, Anna Sartori, Andrea Vieno, Barbara Penzo, Francesco Paolo Russo, Anna Chiara Frigo, Edoardo G Giannini, Manuela Piccinnu, Gian Ludovico Rapaccini, Maria Di Marco, Eugenio Caturelli, Marco Zoli, Rodolfo Sacco, Ciro Celsa, Fabio Marra, Andrea Mega, Maria Guarino, Antonio Gasbarrini, Gianluca Svegliati-Baroni, Francesco Giuseppe Foschi, Andrea Olivani, Alberto Masotto, Pietro Coccoli, Giovanni Raimondo, Francesco Azzaroli, Gianpaolo Vidili, Maurizia Rossana Brunetto, Franco Trevisani and Fabio Farinati on behalf of ITA.LI.CA study group.

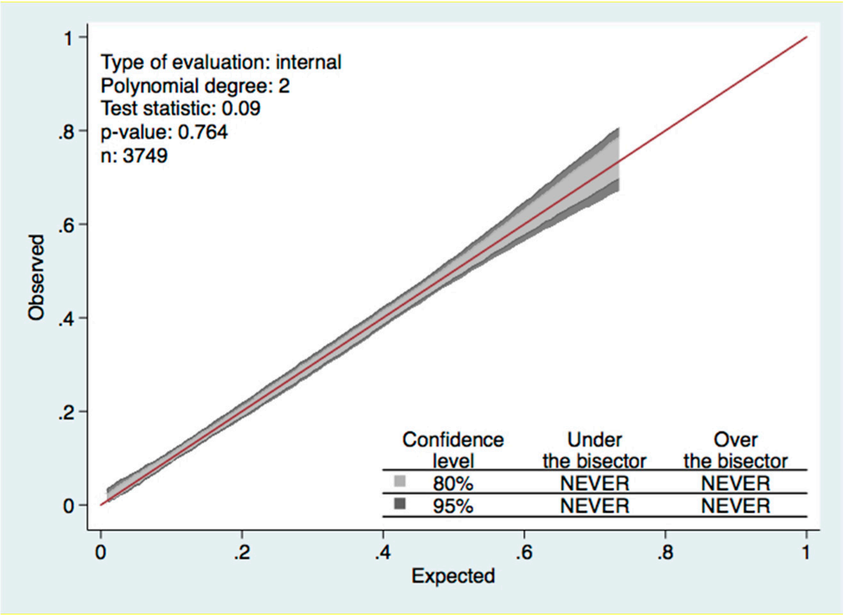

Figure S1. Calibration belt and test of the final multivariable logistic regression model.

Table S1. Bootstrap standard errors and confidence intervals of our multivariable logistic model. One thousand bootstrap replications have been performed.

Variable		Bootstrap OR	Bootstrap 95% CI	Bootstrap Standard Error	*p*
Surveillance	No	Ref	Ref	-	-
	Yes	1.413	1.200–1.664	0.178	<0.0001
Age †		0.989	0.982–0.997	0.004	0.007
MAFLD	No	Ref	Ref	-	-
	Yes	1.299	1.039–1.624	0.148	0.02
CSPH	No	Ref	Ref	-	-
	Yes	0.705	0.579–0.858	0.071	0.001
MELD †		0.877	0.848-0.906	0.015	<0.0001
Treatment	Palliative	Ref	Ref	-	-
	Curative	3.924	3.316–4.645	0.337	<0.0001
Volume	HV	Ref	Ref	-	-
	LV	1.741	1.457–2.079	0.158	<0.0001

† Age and MELD were considered as continuous variables. Palliative treatment: IAT, SOR and other; curative treatments: LR and ABL.Abbreviations: OR, Odds Ratio; CI, confidence interval; aOR, adjusted Odds Ratio; Ref, reference group; MAFLD, metabolic associated fatty liver disease; CSPH, clinically significant portal hypertension; MELD, Model for End-Stage Liver Disease; ITA.LI.CA, Italian Liver Cancer; HV, high-volume institutions; LV, low-volume institutions.
